# Supplementary material for: Adherence to iron and folate supplementation and associated factors among women attending antenatal care in public health facilities at Covid-19 pandemic in Ethiopia
Source: PLOS Glob Public Health. 2023 Jan 18;3(1):e0000825. doi: 10.1371/journal.pgph.0000825 (PMC10021611; doi:10.1371/journal.pgph.0000825)
Supplement: Attachment — Submitted filename: Response to Reviewers 1.docx [file pgph.0000825.s001.docx]

**Response to Reviewers'**

As a general to begin with, we have added additional references

We have changed the paragraphs order as well as the references.

1. Does this manuscript meet PLOS Global Public Health’s [publication criteria](https://journals.plos.org/globalpublichealth/s/criteria-for-publication)? Is the manuscript technically sound, and do the data support the conclusions? The manuscript must describe methodologically and ethically rigorous research with conclusions that are appropriately drawn based on the data presented.

Reviewer #1: Yes

Reviewer #2: Partly

We’ll try to address the issue needed to be incorporated.

 2. Has the statistical analysis been performed appropriately and rigorously?

Reviewer #1: Yes

Reviewer #2: Yes

Thank you.

3. Have the authors made all data underlying the findings in their manuscript fully available (please refer to the Data Availability Statement at the start of the manuscript PDF file)?

The [PLOS Data policy](https://journals.plos.org/globalpublichealth/s/data-availability) requires authors to make all data underlying the findings described in their manuscript fully available without restriction, with rare exception. The data should be provided as part of the manuscript or its supporting information, or deposited to a public repository. For example, in addition to summary statistics, the data points behind means, medians and variance measures should be available. If there are restrictions on publicly sharing data—e.g. participant privacy or use of data from a third party—those must be specified.

Reviewer #1: Yes

Reviewer #2: Yes

Thank you.

4. Is the manuscript presented in an intelligible fashion and written in standard English?

PLOS Global Public Health does not copy edit accepted manuscripts, so the language in submitted articles must be clear, correct, and unambiguous. Any typographical or grammatical errors should be corrected at revision, so please note any specific errors here.

Reviewer #1: Yes

Reviewer #2: No

We have tried to correct typographically/ grammatical errors based on the 1^st^ reviewer comments

 5. Review Comments to the Author
Please use the space provided to explain your answers to the questions above. You may also include additional comments for the author, including concerns about dual publication, research ethics, or publication ethics. (Please upload your review as an attachment if it exceeds 20,000 characters)

It is less than 10, 000

**Reviewer #1:**

This is a very interesting mixed-methods study that aimed to assess adherence to iron and folate supplementation and its associated factors among women attending antenatal care in public health facilities in a city in Ethiopia. Although the importance of the topic, the present study presents several gaps.

1. First of all, the **Background section is very disorganized**. Therefore, for readability, it would be best to approach the content in the following logical sequence: (i) anemia and its consequences for the mother and baby; (ii) the importance of iron and folic acid supplementation; (iii) treatment adherence definition; (iv) proportions of adherence/non-adherence to IFAS worldwide and in the region; (v) exploration of the associated factors; (vi) study rationale; (vii) aims.

We have accepted the comment. First we have corrected the given comments in general and then after we have organized the paragraph based on the logical sequence.
2. **Background section** – The phrase “More than 50% of pregnant women were anemic in Pakistan in 2011(38).” can be removed since the authors address other more up-to-date references.

Removed
3. Also, the correct aim of the study is to assess adherence to iron and folate supplementation and its associated factors among women attending antenatal care in public health facilities in Dire Dawa city administration, Eastern Ethiopia.

Corrected as the comment given.
4. **Methods section** – The authors described the study area very well. However, they did not mention the estimated population size of the city.

We are included estimated population size of the city from the mother document. Based on the 2007 Census conducted by the Central Statistical Agency of Ethiopia (CSA), Dire Dawa has a population of 341,834, of whom 171,461 are men and 170,461 women. 233,224 or 68.23% of the population are urban inhabitants(61).

5. **Methods section** – There was a lack of information about the variables used in the analysis, such as how they were classified; how they were collected (all by self-report?)...

It is already written in the manuscript. Face to face interview for quantitative study to collect data from 308 pregnant women and IDI for key informants/ focal person, health care providers and pregnant mother who were not included under qualitative study. Measurement tools are briefly classified under the operational definition.

6. **Methods section** – In the paragraph: “Appropriate personal protective equipment (PPE) like sanitizer and a facemask was used by the data collectors and provided to the study participants before undergoing data collection. The data collectors used latex disposable gloves while taking information from the ANC card of the study participant in a clinical setting.” it is important to state that those measures were adopted in the face of the COVID-19 pandemic.

Accepted and we have incorporated.
7. **Results section** – “the educational status of pregnant women was 92 (29.9%)”: this information is incomplete.

Secondary and above was missed & we have incorporated besides we have corrected partner educational status from college to secondary. Rewritten the statement.
8. **Results section** – “and 124 (40.3%) of their partner were government employees”: this information differs from the table.

Private is replaced by government
9. **Discussion section** – Several statements are unreferenced:

“This might be due to the fact that educated women have better access to information about iron deficiency anemia, therapy, pregnancy-related risk factors, and their consequences through reading books, the internet, seeking advice from healthcare professionals, and understanding the benefits of the supplement.”;

“This difference can be better elaborated as urban dwellers are obviously living in better infrastructures like good access to transportation and getting to nearby health facilities than rural dwellers. This helps urban dwellers get health advice from health care professionals regularly.”;

“The reason behind this fact is that if a pregnant woman has knowledge about pregnancy related risk factors like birth defects due to folic acid deficiency, it will lead them to minimize the risks by taking IFA supplements. By taking the supplement, she might aware that she can protect herself and her baby from bad pregnancy outcomes.”

For the above mentioned concern, not only three paragraphs written based on the comment given. Almost all paragraphs under discussion part were written in such a way. Thus, we considered the comment in general and made a correction. But I don’t think the reviewer may not miss how to write the discussion. To write the discussion, 1^st^ statement of the finding/result of this study is written and then compared it with the other similar published studies by comparing the finding whether it is similar, different, high, low …. Lastly we write or conclude the possible reason for this similarity or difference based on our assumption thou we don’t need reference for our conclusion. Thus, we have removed the word the fact that since it created confusion. We don’t need references since it is not derived from references, rather they may be the possible reason for the issue. To make it easy to understand, we have separated the statement from the paragraph in order to make it easy to understand.

10. There are some typos in the manuscript.

We try to address this from the 1^st^ page to the end.

**Reviewer #2: The topic described in this study is important and interesting. To improve this manuscript, please read the following suggestions:**

**Abstract**:

1 **Background:** it is better to provide the actual dose than use the ”tab”.

Ok modified

2 **Methods:** the 1st sentence needs to be improved for readability; How was adherence defined?

The 1^st^ sentence is corrected.

But How was adherence defined? Is not part of method rather a background. Thus,

It is also corrected. We have used definition from new reference.

Adherence is the extent to which a patient’s behavior follows corresponds medical instructions given from a health care provider. <https://emocha.com/what-is-medication-adherence/>

3 **Results:** please indicate the high/low maternal education, low/high number of ANC visits, high/low gestational age, with/without knowledge about birth defects were associated with ...; please also indicate what categories were included in the place of residence variable.

The comment raised here is in detail found under table 4 and as well as in the result and discussion part. The listed variables are used to show which variable is significantly associated with IFA supplements generally. Specific factors like Primary education, Urban, >=4 visits, early registration, High knowledge of BD are not written here because the reader can get it in detail under the result and discussion part. The reviewer should have to understand this. It is possible as it is. But for making things smooth I have adjusted as the reviewer comment for the betterment of our work.

We have corrected.

**Background**
Please rearrange the background to increase readability and logic flow.
1 The sentence “However, most pregnant women in the world did not consume the full amount (180 tablets/ 6 months)” needs a reference.

The reference is added

Protection, Promotion and Support of Healthy Maternal, Infant and Young Child Feeding. Iron Deficiency Anaemia (IDA). The Essential Nutrition Actions (ENAs) Approach. September, 2019. <https://motherchildnutrition.org/nutrition-protection-promotion/essential-nutrition-actions/control-of-iron-deficiency-anaemia-ida.html>

2 Why we need “another” in the “Hence, there is a need for another mechanism to measure adherence, which should be relevant, attainable, and reasonable.” is not clear

Another is removed.

3 Please double check the references for “Women are said to have adhered to IFAS if they take the supplement at least 4 days a week. (3–15).”, not all of them can support the statement.

Corrected from this part we have got other references, Thus, we have taken these and used as a new reference 24,40. But for reference 3-15 is changed to 63-73

4 Please provide a reference for the sentence “In Ethiopia, about 74% of women received ANC from a skilled provider at least once for their last birth and four in10 women (43%) had four or more ANC visits for their most recent live birth.”.

Reference 32

CSA and ICF. The 2019 Ethiopia Mini Demographic and Health Survey [Internet]. Addis Ababa, Ethiopia, and Rockville, Maryland, USA: CSA and ICF; 2019 [cited 2020 Jan 24]. Available from: <https://www.unicef.org/ethiopia/reports/2019-ethiopia-mini-demographic-and-health-survey>

5 Please provide a reference for “In Ethiopia, about 74% of women received ANC from a skilled provider at least once for their last birth, and four in 10 women (43%) had four or more ANC visits for their most recent live birth.”

Repeated comment

**Methods:**

1 Please provide more information about the choice of assumption for the calculation of sample size, for example, why did you assume the nonresponse rate to be 10%.

It is known. We can take 5 since we have used CI of 95% or 3 or 4. But we have taken 10 non response rate in order to have a little bit larger sample size. This is scientifically known. Thus, we don’t need to explain each and everything here.

2 Please revise the sample size calculation and sampling method to improve readability.

Corrected

**Results:**

1 The overall structure of the result section needs to be improved. All the information in the result section should be connected and tell a clear story. We have done it.

2 “This finding was supported by a qualitative study” is not clear. Please improve its readability.

We have done it.

**Whole manuscript:**

1. The grammar of the whole draft needs to be revised to improve readability. Most information communicated in this manuscript is not clear enough for the reviewer to provide suggestions with confidence.

Thank you for your comment. We have tried to make the draft readable and easy to understand.
